# Accuracy of Principal and Teacher Knowledge of School District Policies on Sun Protection in California Elementary Schools

**DOI:** 10.5888/pcd15.170342

**Published:** 2018-01-18

**Authors:** David B. Buller, Kim D. Reynolds, Julia Berteletti, Kim Massie, Jeff Ashley, Mary Klein Buller, Richard T. Meenan

**Affiliations:** 1 Klein Buendel, Inc, Golden, Colorado; 2 School of Community & Global Health, Claremont Graduate University, Claremont, California; 3 Sun Safety for Kids, Burbank, California; 4 Kaiser Permanente Center for Health Research, Portland, Oregon

## Abstract

**Introduction:**

Policy is a key aspect of school-based efforts to prevent skin cancer. We explored the extent and accuracy of knowledge among principals and teachers in California public school districts about the elements specified in their district’s written sun safety policy.

**Methods:**

The sample consisted of California public school districts that subscribed to the California School Boards Association, had an elementary school, adopted Board Policy 5141.7 for sun safety, and posted it online. The content of each policy was coded. Principals (n = 118) and teachers (n = 113) in elementary schools were recruited from September 2013 through December 2015 and completed a survey on sun protection policies and practices from January 2014 through April 2016.

**Results:**

Only 38 of 117 principals (32.5%) were aware that their school district had a sun protection policy. A smaller percentage of teachers (13 of 109; 11.9%) than principals were aware of the policy (*F*_108_ = 12.76, *P* < .001). We found greater awareness of the policy among principals and teachers who had more years of experience working in public education (odds ratio [OR] = 1.05, *F*_106_ = 4.71, *P* = .03) and worked in schools with more non-Hispanic white students (OR = 7.65, *F*_109_ = 8.61, *P* = .004) and fewer Hispanic students (OR = 0.28, *F*_109_ = 4.27, *P* = .04).

**Conclusion:**

Policy adoption is an important step in implementing sun safety practices in schools, but districts may need more effective means of informing school principals and teachers of sun safety policies. Implementation will lag without clear understanding of the policy’s content by school personnel.

## Introduction

In 2014, the US Surgeon General issued a call to action to prevent skin cancer (1), citing the high and increasing prevalence (2) and cost of the disease. Unprotected, excessive exposure to solar ultraviolet radiation is the primary risk factor for skin cancer (3–5). The Centers for Disease Control and Prevention (CDC) (6) and the Surgeon General identified sun safety in schools as a priority. Children receive substantial ultraviolet exposure (7), including during outdoor school activities (eg, recess and lunch periods, physical education classes, extracurricular sports). Many children are sunburned annually (8–10), and blistering sunburns in adolescence may elevate lifetime risk for melanoma (5). Schools can design outdoor environments and schedules to reduce ultraviolet exposure and teach lifelong sun protection habits.

Written policy is a key aspect of school-based efforts to prevent skin cancer (1,6,11). Policies can prescribe actions at all levels of school organization (12–15) to improve health outcomes (16–19), including for children who do not choose or are unable to protect themselves (20). However, a policy is successful only if implemented. An essential step in policy implementation is for school personnel, including principals and teachers, to be made aware of the policy.

California had an estimated 7,750 cases of melanoma (23 per 100,000) in 2014 (21,22). It was one of the first states to enact legislation, in 2002, governing sun protection for students (California Education Code Section 35183.5). In 2006, the California School Boards Association (CSBA) recommended a sun safety Sample Board Policy (designated BP 5141.7) consistent with CDC guidelines (6) on rescheduling outdoor activities; increasing use of sun-protective clothing, hats, sunglasses, and sunscreen; supporting education for students, and outreach to families on skin cancer prevention; and allocating resources and increasing accountability for sun safety efforts.

The objective of this study was to explore the extent and accuracy of California elementary school principals’ and teachers’ knowledge about the elements specified in their district’s written school board–approved sun safety policy. The study was restricted to California public school districts that already had a sun safety policy.

## Methods

We conducted an online baseline survey of principals and teachers in schools that were recruited from September 2013 through December 2015 to a parent study, a randomized trial that tested a technical assistance intervention that promoted implementation of sun safety policy by elementary school personnel. Eligible to be included in the sample were public school districts in California that 1) subscribed to the CSBA, 2) had an elementary school, 3) adopted BP 5141.7, and 4) posted their version of BP 5141.7 online.

CSBA staff members provided lists of member districts that had adopted BP 5141.7 in 2012 and 2013. Project staff members contacted by email and telephone the principals at 489 elementary schools in 59 school districts that had adopted BP 5141.7, inviting them to participate in the parent project. The order in which principals at elementary schools were contacted was stratified by location of the elementary school (whether coastal or inland, because these regions differ in temperature and cloud cover); distance from the study’s offices at Claremont Graduate University, Claremont, California (to control project costs, schools closer to Claremont were contacted first); and the number of elementary schools in the district. Elementary schools in larger school districts were contacted first. Recruitment ended when the quota for 118 elementary schools was met for the parent study, which was set based on a priori power estimates for the policy implementation intervention. Of 130 principals who agreed to have their elementary school participate, 12 (9%) did not complete the baseline survey before the recruitment period ended, so the school was not randomized; 359 principals did not respond to the study invitation or refused to participate. One hundred-eighteen principals provided consent for their school to participate and nominated one teacher or staff member (hereinafter referred to simply as “teachers”) who would be involved in implementing sun safety practices at the school. Teachers were contacted individually and invited to complete the baseline survey. If the teacher identified by the principal did not consent, the principal was asked to designate an alternate person.

**Policy collection and coding procedures.** The BP 5141.7 policy documents for districts with participating elementary schools were downloaded by research staff from district websites or from Gamut Online, CSBA’s database of district policies, from September 2013 through December 2015 as schools were recruited to the trial. Research staff members were trained to code the content of each policy document by using a validated coding system (23). Coders recorded the presence of policy content (ie, 0 = not present; 1 = present) in 11 categories covering sun safety components recommended by CDC (6): sunscreen use, ultraviolet-protective clothing, hats, education of students, education of teachers, outdoor shade, scheduling, parent outreach, resource allocation, accountability, and staff modeling. For each category, the strength and intent of the policy components for sun protection of students were recorded but not used in this analysis. The date of adoption and revisions to the policy were recorded. All policies (n = 190) were coded by a single coder. To check inter-rater reliability, 15% (n = 29) of policies were double coded by a second coder; reliabilities for the content and strength score were acceptable (κ > 0.70 [mean = 0.94] [24]). 

**Online survey procedures.** A principal and a teacher at each elementary school completed a survey on school district sun protection policies and school sun safety practices from January 2014 through April 2016. All surveys were completed online by using a survey software package through Inquisit (Millisecond Software). Respondents received an invitation and up to 3 automated email reminders and follow-up telephone calls, if needed, by project staff members. The survey procedures and measures were classified as exempt by the Claremont Graduate University and Western institutional review boards.

Principals and teachers reported whether or not the school district had a policy on sun protection for students. Those who answered yes were presented with a list of policy components (eg, modifying the school schedule to avoid daily periods with high levels of ultraviolet radiation, increasing shade on school grounds, teaching about skin cancer prevention in the classroom) and asked to indicate which components were included in the policy. Respondents also answered items on job characteristics (years in public education and in their current job), skin type (on a scale of 1 to 5, with 1 being the darkest and lowest risk of skin cancer and 5 being the lightest and the highest risk of skin cancer), personal or family history of skin cancer, and demographic characteristics (age, Hispanic ethnicity, race/ethnicity, sex). Respondents also answered questions on attitudes toward sun protection of students, personal sun protection practices, and sunburn history, but these data were not included in this analysis. Skin types categorized as 4 and 5 were designated high-risk skin types. Teachers were also asked which grades they taught and whether they taught health or science or both. Principals and teachers each received $10 for completing the survey.

**School characteristics.** Research staff members obtained information from the state department of education records on location of each school (and then calculated distance from the study office), school size (number of students), and proportion of racial/ethnic minority students, English-learner students, and students in free or reduced-price meal programs.

### Statistical analysis

PROC MEANS and PROC FREQ in SAS version 9.3 (SAS Institute Inc) were used to generate means and percentages to describe the samples of schools, principals, and teachers. Policy knowledge among principals and teachers was compared with the content of the written school district sun safety policy, and principals’ and teachers’ knowledge of each component was classified as accurate or inaccurate. The effects of role (principal vs teacher), demographics characteristics of the principal or teacher, and characteristics of the school on awareness and knowledge of school district sun safety policy were assessed by using multilevel analysis, with individuals (principal or teacher) nested within schools and schools nested within districts. PROC GLIMMIX was used to fit mixed models for logistic regression on awareness of policy and PROC MIXED was used to fit mixed models for linear regression on number of policy elements correctly known. We set α criterion levels at .05 (2-tailed).

## Results

The elementary schools were widely distributed around Claremont, California (Table 1). Schools enrolled on average 564.6 students; 54.5% were Hispanic and 24.0% were non-Hispanic white. Approximately one-quarter (27.8%) of students were English learners, and 64.1% received free or reduced-price meals. We found no significant differences between participating and nonparticipating schools (n = 371) in distance from study office, number of students, race/ethnicity, and or other student characteristics. Principals had worked on average 21.1 years in public education but had served as principals in their current school district for 4.6 years on average (and worked in the current school district for 11.4 years on average). Principals were aged on average 47.8 years; 21.2% were Hispanic, 69.5% were non-Hispanic white; and 72.0% were women. Approximately one-third (34.8%) of principals had a high-risk skin type, and 39.0% reported they or a family member had been diagnosed with skin cancer.

**Table 1:** Profile of the Samples of Elementary Schools (n = 118), Principals (n = 118), and Teachers (n = 113) Who Participated in Study of Knowledge of the School District Written Policy on Sun Protection, California Public School Districts, January 2014–April 2016^a^

Characteristic	Mean (SD) or %
**Schools**	
Distance from study office in Claremont, California, miles^b^	133.6 (180.1)
Total no. of students enrolled in school	564.6 (215.6)
English-learner students, %	27.8 (17.0)
Fluent English-proficient students, %	8.7 (6.6)
Students in free or reduced-price meal program, %	64.1 (28.8)
African American students, %	6.4 (7.6)
American Indian or Alaska Native students, %	1.7 (7.3)
Asian students, %	6.5 (12.8)
Filipino students, %	1.6 (2.7)
Hispanic or Latino students, %	54.5 (26.4)
Pacific Islander students, %	0.5 (1.1)
Non-Hispanic white students, %	24.0 (22.3)
Students identifying as ≥2 races/ethnicities	3.6 (3.3)
**Principals**	
**No. of years working . . .**	
In public education in any position	21.1 (7.1)
As a principal in current school district	4.6 (4.0)
In current school district	11.4 (9.2)
**Skin type**	
1 (Darkest skin/lowest risk)	28.7
2	28.7
3	25.2
4	17.4
5 (Lightest skin/highest risk)	17.4
**Age, y**	47.8 (7.7)
**Personal or family history of skin cancer**	39.0
**Hispanic ethnicity**	21.2
**Race**	
American Indian/Alaska Native	3.4
Asian	0.8
Black/African American	8.5
Native Hawaiian/Other Pacific Islander	0.8
Non-Hispanic white	69.5
More than one race/ethnicity	5.1
Prefer not to answer	3.4
None of these	8.5
**Sex**	
Female	72.0
Male	27.1
Prefer not to answer	0.9
**Teachers**	
**No. of years working . . . **	
In public education in any position	14.3 (7.3)
As a teacher in current school district	11.6 (7.5)
**Grade(s) currently taught^c^**	
Kindergarten	17.0
Grade 1	17.9
Grade 2	18.8
Grade 3	21.4
Grade 4	21.4
Grade 5	18.8
Grade 6	9.8
Grade 7	2.7
Grade 8	1.8
No specific grade	45.1
**Teach health or science curriculum**	
No	58.0
Yes, health	7.1
Yes, science	17.9
Yes, health and science	17.0
**Skin type**	
1 (Darkest skin/lowest risk)	26.4
2	27.3
3	22.7
4	20.9
5 (Lightest skin/highest risk)	2.7
**Age, y**	43.4 (10.0)
**Personal or family history of skin cancer**	42.5
**Hispanic ethnicity**	22.1
**Race**	
American Indian/Alaska Native	4.4
Asian	7.1
Black/African American	4.4
Native Hawaiian/Other Pacific Islander	2.7
Non-Hispanic white	66.4
More than one race/ethnicity	3.5
Prefer not to answer	2.6
None of these	8.9
**Sex**	
Female	86.7
Male	13.3
Prefer not to answer	0

a Sample consisted of California public school districts that subscribed to the California School Boards Association, had an elementary school, adopted Board Policy 5141.7 for sun safety, and posted it online from September 2013 through December 2015. Participants completed surveys from January 2014 through April 2016.

b Median, 42 miles; range, 2–809 miles; interquartile range, 30–96 miles.

c Teachers could select more than one grade level.

In the 118 schools, 113 teachers (96%) completed the survey. They had less experience than principals in public education, averaging 14.3 years, but they had worked a similar amount of time in their school districts at 11.6 years on average. Nearly half (45.1%) of teachers did not teach a specific grade; those who did were widely distributed over grades kindergarten through grade 5. Also, 42.0% of teachers taught health or science or both curricula. Teachers were younger (43.4 years on average) than principals, and the percentage of teachers who were women (86.7%) was greater than the percentage of principals who were women. Approximately one-fifth (22.1%) of teachers were Hispanic, and 66.4% were non-Hispanic white. One-quarter (23.6%) of teachers had high-risk skin types, and 42.5% had a personal or family history of skin cancer.

### Awareness of school district sun safety policy

Of 117 principals who answered the policy awareness item, 38 (32.5%) were correctly aware that their school district had a sun protection policy; 79 principals did not know the district had a policy. Even fewer teachers (13 of 109 [11.9%] who answered the question) were aware of the policy; 96 teachers did not know the district had a policy. Mixed model logistic regression analysis confirmed that principals were more aware of the policy than teachers were (*F*_108_ = 12.76, *P* < .001). In addition, we found greater awareness of the sun protection policy among principals and teachers who had more years of experience working in public education (odds ratio [OR] = 1.05,* F*_106_ = 4.71, *P* = .03) and were in schools with a larger proportion of non-Hispanic white students (OR = 7.65, *F*_109_ = 8.61, *P* = .004) and a smaller proportion of Hispanic students (OR = 0.28, *F*_109_ = 4.27, *P* = .04).

### Awareness of policy content

Among the principals and teachers who were aware of their district’s policy on sun protection, knowledge of its content elements varied (Table 2). When principals or teachers had inaccurate knowledge, they tended to report that the policy did not address a particular component of sun safety when it actually did. Approximately three-quarters of principals were aware that the policy addressed personal protection of students (ie, use of sunscreen, 68.4%; use of protective clothing, 71.1%, and use of hats, 76.3%). Most principals were correctly aware that the policy did not cover training teachers (84.2%) or allocating district resources for sun protection (91.9%). Approximately half of principals had accurate knowledge of whether the policy addressed educating students (44.8%) and communicating with parents on sun safety (54.0%) and adjusting schedules for students to be outdoors outside of midday hours when levels of ultraviolet radiation is highest (48.6%). Principals had the greatest percentage of inaccurate knowledge on whether the policy addressed providing shade on school grounds (60.5%) and encouraged school staff members to model sun safety to students (59.5%).

**Table 2:** Percentage of Correct and Incorrect Knowledge of the School District Written Policy on Sun Protection Among Elementary School Principals (N = 38) and Teachers (N = 13) Who Were Aware of the Policy, California Public School Districts, January 2014–April 2016^a^

Policy Component	Accurate Policy Knowledge^b^, %			Inaccurate Policy Knowledge^c^, %		
	Addressed in Policy	Not Addressed in Policy	Total	Addressed in Policy	Not Addressed in Policy	Total
**Principals**						
Sunscreen use	68.4	5.3	73.7	2.6	23.7	26.3
Ultraviolet-protective clothing use	71.1	0	71.1	2.6	26.3	28.9
Hat use	76.3	0	76.3	2.6	21.1	23.7
Educate students on sun safety	23.7	21.1	44.8	10.5	44.7	55.2
Educate teachers on sun safety	0	84.2	84.2	15.8	0	15.8
Provide outdoor shade	23.7	15.8	39.5	2.6	57.9	60.5
Adjust schedules so outdoor activities avoid midday	35.1	13.5	48.6	2.7	48.7	51.4
Communication with parents about sun safety	32.4	21.6	54.0	8.1	37.9	46.0
Allocate resources for sun safety	0	91.9	91.9	8.1	0	8.1
Encourage staff to model sun safety	32.4	8.1	40.5	2.7	56.8	59.5
**Teachers**						
Sunscreen use	38.5	7.7	46.2	7.7	46.1	53.8
Ultraviolet-protective clothing use	53.8	0	53.8	0	46.2	46.2
Hat use	53.8	0	53.8	0	46.2	46.2
Educate students on sun safety	15.4	30.8	46.2	7.7	46.2	53.9
Educate teachers on sun safety	0	92.3	92.3	7.7	0	7.7
Provide outdoor shade	30.8	23.1	53.9	7.7	38.4	46.2
Adjust schedules so outdoor activities avoid midday	38.5	15.4	53.9	0	46.1	46.1
Communication with parents about sun safety	23.1	23.1	46.2	15.4	38.4	53.8
Allocate resources for sun safety	0	92.3	92.3	7.7	0	7.7
Encourage staff to model sun safety	23.1	0	23.1	7.7	69.2	76.9

a Sample consisted of California public school districts that subscribed to the California School Boards Association, had an elementary school, adopted Board Policy 5141.7 for sun safety, and posted it online from September 2013 through December 2015. Participants completed surveys from January 2014 through April 2016.

b Accurate policy knowledge means that school district policy addressed policy component and principal said the component was addressed in the policy, or school district policy did not address policy component and principal said the component was not addressed in the policy.

c Inaccurate policy knowledge means that school district policy addressed policy component and principal said the component was not addressed in the policy, or school district policy did not address policy component and principal said the component was addressed in the policy.

Compared with principals, teachers were much less aware of the content of the district sun safety policy, except for knowing that no provision existed for educating teachers (92.3%) or allocating resources for sun safety (92.3%) (Table 2). Approximately half of the teachers accurately reported whether the policy addressed personal sun protection of students while at school (ie, use of sunscreen, 46.2%; use of protective clothing, 53.8%, and use of hats, 53.8%), educating students on sun safety (46.2%), providing outdoor shade at the school (53.9%), adjusting outdoor activity schedules to avoid midday hours (53.9%), and communicating with parents about sun safety (46.2%). Only 23.1% of teachers knew the policy encouraged school staff to model sun safety to students.

Mixed model linear regression confirmed that principals had accurate knowledge of more content components in the district sun safety policy than teachers (*F*_108_ = 15.98, *P* < .001). In addition, we found greater awareness of the school policy content among principals and teachers who were older (β = 0.037; standard error [SE], 0.015; *F*_98_ = 6.30; *P* = .01), who had more years of experience working in public education (β = 0.040; SE, 0.016; *F*_106_ = 6.08; *P* = .015), who were in schools with a larger proportion of non-Hispanic white students (β = 1.950; SE, 0.580; *F*_109_ = 11.31; *P* = .001), and who were in schools with a smaller proportion of Hispanic students (β = −1.382; SE, 0.498; *F*_109_ = 7.70; *P* = .007), English-learner students (β = −1.802; SE, 0.779, *F*_107_ = 5.35; *P* = .02), and students receiving free or reduced-price meals (β = −1.024; SE, 0.468; *F*_109_ = 4.80; *P* = .03).

## Discussion

In California school districts that had adopted a sun safety policy, a survey of elementary school principals and teachers indicated a low level of awareness of the sun protection elements in their district policy. Most principals and teachers did not know whether or not their district had a sun safety policy. Among those who were aware of the policy, many had incorrect knowledge of its contents. The low level of understanding of the school district’s sun protection policy may reflect a feeling by school personnel that sun safety is a low-priority health and safety topic relative to other issues such as tobacco and substance use and nutrition. Principals and teachers may also consider health and safety issues to be a lower priority than curricular and testing issues.

The greater awareness of the policy among principals than among teachers also may indicate shortcomings in the way many school districts communicate new policies to school personnel. Principals may be more aware of district policies than teachers because they are in a leadership position and thus more likely to be informed about policy changes by district administrators. Districts may rely on principals in turn to communicate policies to teachers, and some principals may do this more effectively than others. In addition, some principals may have more responsibility than teachers for implementing policies that affect the health and safety of students, especially when implementation requires school-wide actions (eg, adjusting outdoor schedules and constructing shade structures) rather than class-wide or grade-wide actions.

Principals and teachers were more knowledgeable about how the elements of the policy pertained to personal protection practices for students (ie, use of sunscreen, protective clothing, and hats) than to the education of students or modifications to the school environment and procedures (ie, provision of shade, adjustment of school schedules, and modeling of sun safety by school staff members). School personnel may consider sun protection to be a personal choice and the responsibility of students and their parents and thus would expect sun protection policy to address students’ personal protection practices. Also, these personal protection practices are covered by state legislation, so awareness of the legislation may have elevated their awareness of their school district’s policy.

In addition to parents’ and students’ personal responsibility for sun protection, more efforts appear to be needed to raise awareness among school personnel about their role and responsibility in implementing modifications to the school environment and procedures when these elements are expressed in the district policy. Environmental and procedural changes are enduring, apply to all students, and offer a certain level of protection for students who do not practice personal sun protection for whatever reason.

That the teachers surveyed in this study were largely unaware of the sun protection policy was surprising, even though principals identified these individuals as someone at the school who would assist in implementing sun safety practices. Teachers may be more likely to learn about policies that affect the curriculum, their classroom procedures, or their own jobs. Teachers did appear to be more aware than principals about whether the policies required them to receive training in sun safety.

The sociodemographic characteristics of the student population at the schools appeared to be associated with principals’ and teachers’ knowledge of their district’s sun protection policy. Principals and teachers at schools with a greater percentage of non-Hispanic white students and affluent students (ie, students who were not participating in a free or reduced-price lunch program) and a smaller percentage of Hispanic and English-learner students were more aware of the sun protection policy and its contents. This finding may reflect a general knowledge that skin cancer is more prevalent among non-Hispanic white people than among Hispanic people. However, the prevalence of skin cancer is increasing in Hispanic populations (25), and school personnel should be made aware of this fact and the relevance of skin cancer prevention to Hispanic students. The finding that school personnel who had more experience working in public education were more informed about the sun protection policies may be attributed to principals being older than teachers. School personnel with more experience may have more extensive professional communication networks that provided them with more information about the new policies than less experienced and younger school personnel. This finding calls for senior administrators to initiate strategies that actively communicate new policies throughout the school districts to make sure the new policies are disseminated across all personnel regardless of the depth of their professional contacts and position in the district’s leadership.

The study had several strengths and limitations. Strengths are that the sample of schools was large and the presence and content of district policy was obtained from coding of written policy documents. However, the study was limited to a single state’s elementary schools, limiting generalizability. The survey items did not assess details of sun protection (eg, sunscreen sun protection factor, topics in student instruction) and relied on self-report. Efforts and resources devoted by school districts to publicize the policies to principals and teachers were not assessed.

The US Surgeon General’s call to action to prevent skin cancer states that “[w]e must act with urgency to stop the ever-increasing incidence of skin cancers in the United States.” The call to action identifies schools as essential partners in this effort. Schools are urged to increase the availability of sun protection in educational settings, support skin cancer prevention education, and support inclusion of sun protection in school policies, construction of school facilities, and school curricula. Policy adoption by an elected school district board may be a necessary first step toward implementing other sun safety practices and procedures in schools. However, the knowledge gap among elementary school principals and teachers in their awareness of district sun safety policies suggests a need for improved communication of the policy to principals and teachers as well as encouragement and support for policy implementation so that sun protection can be improved in elementary schools.
